# Transcript Levels of Aldo-Keto Reductase Family 1 Subfamily C (AKR1C) Are Increased in Prostate Tissue of Patients with Type 2 Diabetes

**DOI:** 10.3390/jpm10030124

**Published:** 2020-09-12

**Authors:** Andras Franko, Lucia Berti, Jörg Hennenlotter, Steffen Rausch, Marcus O. Scharpf, Martin Hrabĕ de Angelis, Arnulf Stenzl, Andreas L. Birkenfeld, Andreas Peter, Stefan Z. Lutz, Hans-Ulrich Häring, Martin Heni

**Affiliations:** 1 Department of Internal Medicine IV, Division of Endocrinology, Diabetology and Nephrology, University Hospital Tübingen, 72076 Tübingen, Germany; andras.franko@med.uni-tuebingen.de (A.F.); andreas.birkenfeld@med.uni-tuebingen.de (A.L.B.); s.lutz@bad-sebastiansweiler.de (S.Z.L.); hans-ulrich.haering@med.uni-tuebingen.de (H.-U.H.); 2 Institute for Diabetes Research and Metabolic Diseases, Helmholtz Centre Munich, University of Tübingen, 72076 Tübingen, Germany; lucia.berti@helmholtz-muenchen.de; 3 German Center for Diabetes Research (DZD), 85764 Neuherberg, Germany; hrabe@helmholtz-muenchen.de; 4 Department of Urology, University Hospital Tübingen, 72076 Tübingen, Germany; joerg.hennenlotter@med.uni-tuebingen.de (J.H.); steffen.rausch@med.uni-tuebingen.de (S.R.); arnulf.stenzl@med.uni-tuebingen.de (A.S.); 5 Institute of Pathology, University Hospital Tübingen, 72076 Tübingen, Germany; marcus.scharpf@med.uni-tuebingen.de; 6 Institute of Experimental Genetics, Helmholtz Zentrum München, German Research Center for Environmental Health, 85764 Neuherberg, Germany; 7 Institute for Clinical Chemistry and Pathobiochemistry, Department for Diagnostic Laboratory Medicine, University Hospital Tübingen, 72076 Tübingen, Germany; andreas.peter@med.uni-tuebingen.de (A.P.); 8 Clinic for Geriatric and Orthopedic Rehabilitation Bad Sebastiansweiler, 72116 Mössingen, Germany

**Keywords:** prostate cancer, diabetes, aldo-keto reductase family 1, HIF1a, NFkB

## Abstract

Aldo-keto reductase family 1 (AKR1) enzymes play a crucial role in diabetic complications. Since type 2 diabetes (T2D) is associated with cancer progression, we investigated the impact of diabetes on *AKR1* gene expression in the context of prostate cancer (PCa) development. In this study, we analyzed benign (BEN) prostate and PCa tissue of patients with and without T2D. Furthermore, to replicate hyperglycemia in vitro, we treated the prostate adenocarcinoma cell line PC3 with increasing glucose concentrations. Gene expression was quantified using real-time qPCR. In the prostate tissue of patients with T2D, *AKR1C1* and *AKR1C2* transcripts were higher compared to samples of patients without diabetes. In PC3 cells, high glucose treatment induced the gene expression levels of *AKR1C1*, *C2,* and *C3*. Furthermore, both in human tissue and in PC3 cells, the transcript levels of *AKR1C1, C2,* and *C3* showed positive associations with oncogenes, which are involved in proliferation processes and HIF1α and NFκB pathways. These results indicate that in the prostate glands of patients with T2D, hyperglycemia could play a pivotal role by inducing the expression of *AKR1C1, C2,* and *C3*. The higher transcript level of *AKR1C* was furthermore associated with upregulated HIF1α and NFκB pathways, which are major drivers of PCa carcinogenesis.

## 1. Introduction

Patients with type 2 diabetes (T2D) have a high risk of developing diabetes-driven complications, including retinopathy, neuropathy, and nephropathy [1]. Various drugs show beneficial effects in ameliorating diabetes and its complications. The aldo-keto reductase (AKR) superfamily is one of the most promising drug targets for alleviating diabetic complications [2]. The human AKR superfamily consists of 13 multifunctional AKR proteins, all of which are NAD(P)(H)-dependent oxidoreductases [3]. These enzymes are involved in phase 1 metabolism of carbonyl substrates, including sugars, keto-steroids, and keto-prostaglandins [4]. AKR1B1 (aldose reductase) is crucial for the polyol pathway, where glucose is reduced to sorbitol, which, in turn, is metabolized to fructose [5]. In diabetic conditions, hyperglycemia results in elevated intracellular sorbitol and fructose contents; these evoke osmotic stress and subsequent tissue damage [4]. The application of AKR1B1 inhibitors could prevent or delay hyperglycemia-induced pathologies such as nephropathy, neuropathy, and retinopathy [2]. The molecular pathways that can be activated via AKR1B1 under hyperglycemic conditions include oxidative stress and inflammatory processes. Applications of AKR1B1 inhibitors were, in fact, reported to attenuate both pathways [4].

In addition to classical diabetes complications, T2D is a further risk factor for cancer onset and progression [6]. However, most patients with T2D show a reduced risk of developing PCa [7], although if PCa occurs, these patients frequently showed a more aggressive cancer phenotype and they had markedly lower survival rates than men without diabetes [8,9]. Due to the synergism between T2D and cancer, we sought to determine whether AKR1 proteins could also be implicated in PCa progression in men with T2D. The progression of steroid-dependent cancers such as PCa, as well as breast and endometrial cancers, are already known to be characterized by the altered expression of *AKR1* superfamily genes, in particular, the *AKR1C* subfamily [10]. AKR1C1, C2, and C3 proteins regulate steroid hormone signaling [10] and play a key role in cancer growth, metastasis, and apoptosis [11]. However, the precise mechanisms that cause increased AKR1C protein levels or activities during the onset/progression of PCa have yet to be determined.

Given that AKR1 members were postulated to control oxidative stress and inflammatory processes in the case of T2D, we examined the expression of inflammatory genes and their association with the main AKR1 members in human prostate tissue. Since we were interested in tumor progression, we analyzed both benign (BEN) prostate tissue and PCa samples. To determine the effects of diabetes, we compared samples of patients with and without T2D. Furthermore, to replicate hyperglycemia and PCa conditions in vitro, we treated PC3 prostate cancer cells with ascending glucose concentrations for a prolonged period of time.

Here, we report that the diabetic status in human prostate tissue, together with prolonged hyperglycemic conditions in PC3 cells, induced the gene expression of *AKR1C1*, *C2,* and *C3*. Furthermore, the expression of *AKR1C* members correlated positively with proliferation markers, as well as with hypoxia-inducible factor 1 subunit alpha (HIF1α), and nuclear factor of kappa light chain gene enhancer in B-cells (NFκB) pathways.

## 2. Results

**Figure 1:**
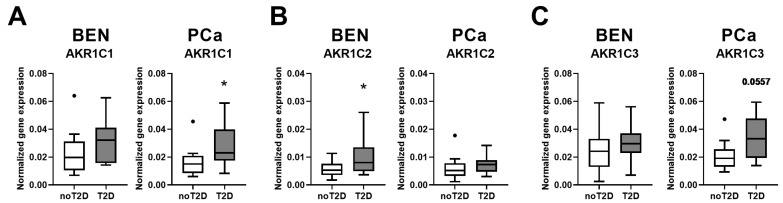
Gene expression levels of *AKR1C1*, *AKR1C2,* and *AKR1C3* were measured in benign (BEN) prostate (*n* = 17‒17) and prostate cancer (PCa) (*n* = 11‒11) tissues of PCa patients with (T2D) and patients without (noT2D) type 2 diabetes. Data are shown as Tukey box plots. Dots denote individual values, which were higher than the sum of the 75th percentile plus 1.5-times inter-quartile range. Statistical significance was calculated using Mann–Whitney tests and considered as *p* < 0.05. * *p* < 0.05. The *p*-value for *AKR1C3* PCa data is indicated.

To determine the impact of diabetes in the progression of prostate cancer (PCa), we analyzed specimens from age- and body mass index (BMI)-matched human cohorts and investigated benign (BEN) prostate and PCa tissues of patients with and without type 2 diabetes (T2D). The following four groups were studied: BEN noT2D (*n* = 17); BEN T2D (*n* = 17); PCa noT2D (*n* = 11); PCa T2D (*n* = 11) (Table S1). To analyze PCa patients at similar tumor stages, only patients with a Gleason score of 7a and 7b were selected. Since AKR family members have been reported to be major players in diabetic complications [4], we aimed to test whether these proteins are also involved in PCa progression in patients with T2D. We, therefore, examined the main AKR members in prostate tissue. The gene expression levels of *AKR1B1,* which plays a key role in hyperglycemia-induced diabetic complications [2], remained unchanged in prostate samples of patients with T2D (Figure S1A). However, we observed major changes in the gene expression of the AKR1C family members. The mRNA level of *AKR1C1* was significantly higher in PCa of the T2D group than in patients without diabetes (Figure 1A). In the BEN T2D group, we observed higher gene expression level of *AKR1C2* than in the group without diabetes (Figure 1B). The expression level of *AKR1C3* tended to be higher (*p* = 0.0557) in the PCa T2D group than in samples from patients without diabetes (Figure 1C).

These results suggest that the gene expression of *AKR1C* members is higher in the prostate of men with T2D. To examine whether *AKR1C* downstream pathways are possibly also altered in diabetes, we investigated further genes involved in oncogenic processes such as proliferation and HIF1α and NFκB pathways (Table S2). The RELA proto-oncogene, NFκB subunit (*RELA)*, showed a significantly higher transcript level in the BEN T2D group than in BEN noT2D tissue (Table S2). In comparison to the PCa noT2D group, the mRNA level of the proliferation marker proliferating cell nuclear antigen (PCNA) in PCa, T2D tissue had increased significantly (Table S2). None of the other genes analyzed showed significant differences between patients with and without T2D (Table S2). We next tested by multiple linear regression analyses whether the gene expression level of *AKR1C* members and the downstream oncogenic pathways are associated. The gene expressions of *AKR1C1, AKR1C2,* and *AKR1C3* showed positive correlations with each other in prostate tissue (Table 1).

**Figure 2:**
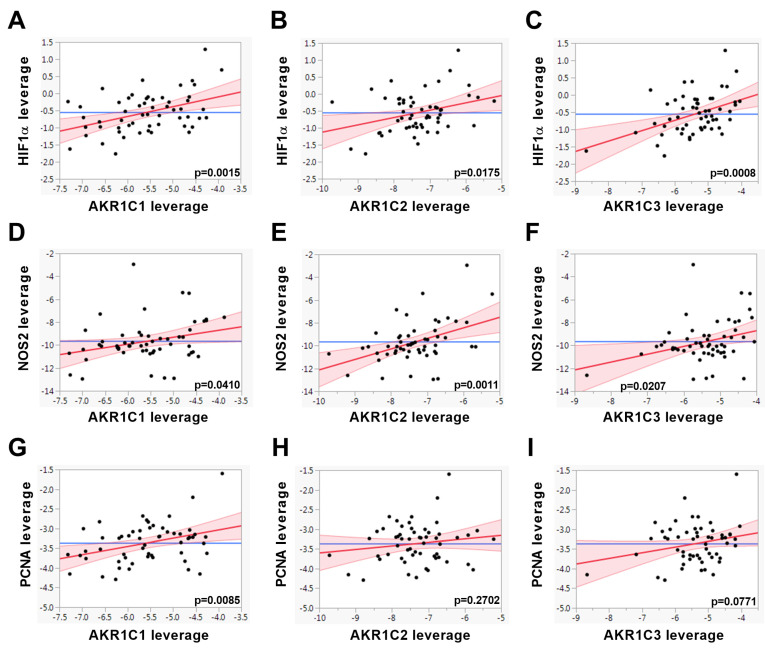
The gene expression levels of *AKR1C1, AKR1C2,* and *AKR1C3* were associated with the genes indicated using multiple linear regression models adjusted for age and BMI in benign (BEN) prostate and prostate cancer (PCa) tissues of PCa patients with and without T2D (*n* = 56) and are shown as leverage plots. Statistical significance was considered as *p* < 0.05. The *p*-values are indicated in the figures.

Furthermore, we found significant positive associations between the expressions of *AKR1C* members and genes involved in proliferation processes and HIF1α and NFκB pathways (Table 1 and Figure 2). These results indicate that the elevated expression of *AKR1C1, AKR1C2,* and *AKR1C3* in prostate samples of patients with T2D is associated with active oncogenic pathways.

**Figure 3:**
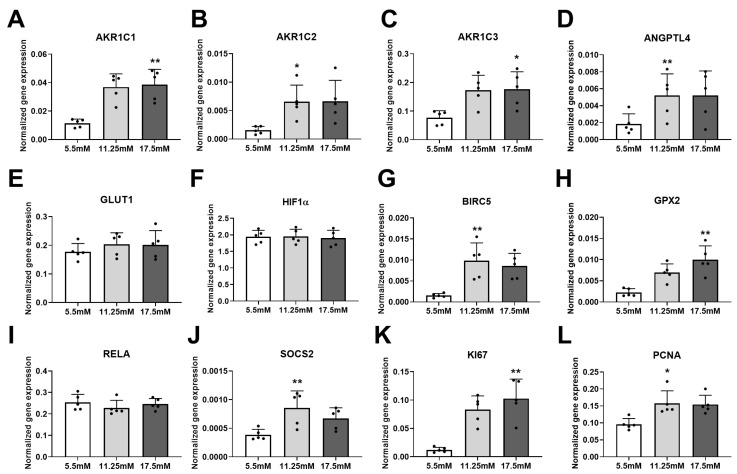
Gene expressions were measured in 5.5, 11.25, and 17.5 mM glucose-treated PC3 cells (*n* = 5 independent experiments). Dots denote individual values. Statistical significance was compared to 5.5 mM control samples using a paired Friedman test with Dunn’s multiple comparisons. Statistical significance was considered as *p* < 0.05 and indicated as * *p* < 0.05 and ** *p* < 0.01.

In patients with T2D, the increased blood glucose levels might be one of the major drivers activating gene expression of *AKR1C* members in PCa tissue. To examine whether hyperglycemia per se can influence the expression of *AKR1C* members, we used the adenocarcinoma cell line PC3 and treated these cells with 5.5, 11.25, and 17.5 mM glucose for 72 h. Hyperglycemia was indeed found to induce the mRNA levels of *AKR1C1, AKR1C2,* and *AKR1C3* (Figure 3A–C). Glucose treatment did not alter the expression of *AKR1B1* (Figure S1B). In both human prostate tissue and PC3 cells, the transcript of the liver-specific *AKR1C4* was not detected (data not shown). Furthermore, the expression level of several genes that are involved in both HIF1α (Figure 3D–F) and NFκB (Figure 3G–J) pathways, as well as in proliferation processes (Figure 3K,L), were induced when the glucose concentration in the cell culture medium was increased.

**Figure 4:**
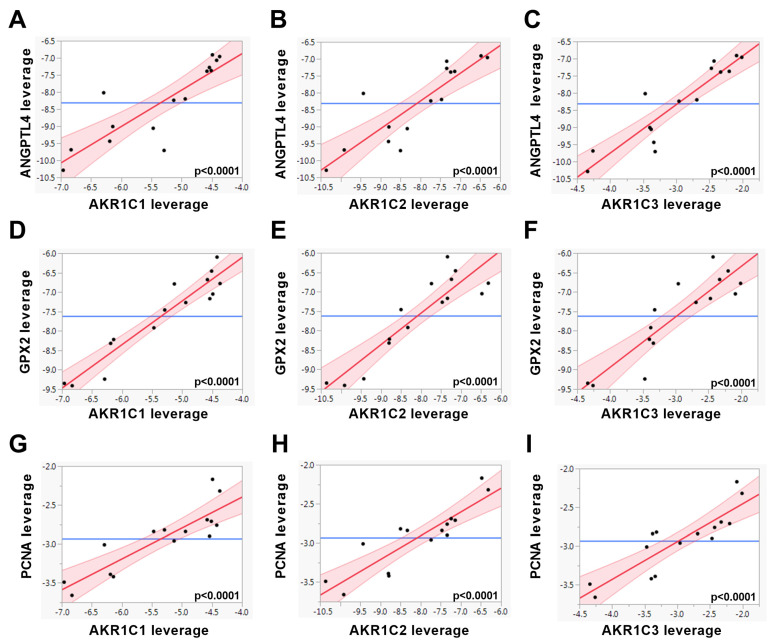
The gene expression levels of *AKR1C1, AKR1C2,* and *AKR1C3* were associated with the genes indicated using multiple linear regression models in 5.5, 11.25, and 17.5 mM glucose-treated PC3 cells (*n* = 15) and are shown as leverage plots. Statistical significance was considered as *p* < 0.05. The *p*-values are indicated in the figure.

In PC3 cells, multiple linear regression analysis revealed that the majority of the oncogenic genes were associated positively with the expressions of *AKR1C* transcripts (Table 2 and Figure 4). These results indicate that in prostate samples of patients with T2D, hyperglycemia could be a driver of the higher expression of those *AKR1C* members associated with oncogenic alterations, such as proliferation processes and HIF1α and NFκB pathways.

## 3. Discussion

The impact of T2D on PCa is complex [7], and the relationship is influenced by numerous factors that are difficult to assess individually [12]. While a large number of studies have reported a lower PCa risk in patients with T2D [13], they are characterized by more advanced PCa than patients with normal glucose metabolism [8,14]. On the one hand, many cancer therapies, including the treatment of PCa with androgen deprivation therapy (ADT), increase the risk for metabolic syndrome, cardiovascular disease, and diabetes [6,15,16]. On the other hand, several diabetes therapies with insulin-lowering properties appear to be beneficial, particularly for patients with advanced PCa [17]. Therapy options that could influence both diabetes and cancer are, therefore, of particular interest. High glucose is known to activate the inflammatory pathways, which are also implicated in diabetic complications [4]. Indeed, the inhibition of aldo-keto reductase family 1 member B1 (AKR1B1, aldose reductase) attenuated inflammation as well as diabetes-induced complications [2]. Furthermore, AKR1B1 could also play an important role in cancer progression, since higher AKR1B1 expression and activity were observed in breast, colon, and lung cancers [18]. In contrast to AKR1B1, the AKR1C subfamily showed no or only very low enzymatic activities that reduced advanced glycation end products, i.e., key regulators of diabetic complications [19]. These results suggest that AKR1C enzymes are probably not central to the development of diabetic complications. Nevertheless, numerous studies have reported that AKR1C members are implicated in the carcinogenesis of PCa [11].

To determine whether AKR1 family members are involved in the development of PCa in diabetes, we analyzed benign (BEN) and PCa tissues of PCa patients with and without T2D. Our data showed that the mRNA level of *AKR1B1* was not altered in diabetes. Furthermore, we investigated the gene expressions of AKR1C members, which are the main steroid-transforming AKR1s in the prostate [3]. We observed that the gene expression levels of *AKR1C1* and *C2* had increased significantly and that the *AKR1C3* mRNA level tended to be higher in the prostate tissue of patients with T2D than in samples of patients without diabetes.

Since T2D is often accompanied by further disarrangements, including obesity, metabolic syndrome, dysregulated lipid metabolism, hyperinsulinemia, and hyperglycemia [20], it is difficult to pinpoint which metabolic disarrangement is the main trigger for cancer progression in PCa patients with T2D. Insulin was recently shown to directly enhance the migration of PCa cell lines such as LNCaP, 22RV1, and DU145 [21]. In addition, high glucose treatment in PC3 cells enhanced insulin-driven oncogenic processes [22]. To replicate hyperglycemia in vitro, PC3 cells were treated with increasing glucose concentration for 72 h. The transcripts of *AKR1C1*, *C2,* and *C3* were significantly upregulated in high glucose-treated PC3 cells. We quantified gene expressions of two major oncogenic regulators (HIF1α and RELA (NFκB subunit) transcripts) in combination with downstream oncogenic genes such as *ANGPTL4* and *GLUT1*, both of which are HIF1α target genes [23,24], and *BIRC5, GPX2, NOS2,* and *SOCS2,* which are NFκB target genes [23,25,26]. Furthermore, we measured the transcripts of genes involved in cell proliferation (*MKI67* and *PCNA*). In both human prostate tissue and PC3 cells, the gene expressions of *AKR1C1*, *C2,* and *C3* showed positive associations with those genes involved in proliferation processes and HIF1α and NFκB pathways. These results indicate that in patients with T2D, hyperglycemia induces the expressions of *AKR1C1*, *C2,* and *C3* genes, thereby enhancing proliferation and activating HIF1α and NFκB pathways.

AKR1C proteins are thought to be involved in the progression of various tumors, including breast, endometrial, and prostate cancers [27]. Previous studies reported an increase in the *AKR1C3* transcript level in both primary PCa and advanced castrate-resistant prostate cancer (CRPCa) [28,29]. Even if the expression pattern of *AKR1C1* and *C2* is influenced by the cancer state (primary PCa vs. advanced CRPCa), the molecular mechanism behind this difference has not yet been elucidated [30]. A comparison of primary PCa and BEN controls revealed lower *AKR1C1* and *C2* mRNA levels [31,32,33]. However, in androgen-independent PCa bone marrow metastases, higher gene expressions were described for *AKR1C1* and *C2* than for primary PCa [29]. These results suggest that the elevated gene expressions of *AKR1C1*, *C2,* and *C3* observed in the prostate tissue of patients with T2D are reminiscent of the expression pattern of an advanced-stage CRPCa.

What are the biological consequences of the diabetes-induced *AKR1C* expressions in terms of carcinogenesis? The multifunctional nature of AKR1C enzymes may provide an answer to this question. AKR1C family members show 3-keto, 17-keto, and 20-ketosteroid reductase and 3α-, 17α-, and 20α-hydroxysteroid oxidase activity and are involved in biosynthetic, metabolic, and detoxification processes [11]. Furthermore, AKR1C enzymes were shown to regulate androgen, estrogen, and progesterone signaling [10]. In the prostate tissue, AKR1C proteins play a pivotal role in the local steroid/androgen synthesis and degradation. They have additional “nonclassical” functions such as detoxification processes and prostaglandin synthesis [30].

ADT is frequently used to treat locally recurrent and advanced PCa. However, with ADT, PCa could modify its metabolism and activate alternate biochemical pathways to increase intratumoral androgen levels [30]. Thereby, dysregulated androgen receptor signaling is one of the major drivers of CRPCa [34]. The AKR1C3 enzyme catalyzes the biosynthesis of major androgen receptor ligands (testosterone (T) and dihydrotestosterone (DHT)) [35]. In addition to its function in the synthesis of these androgens, AKR1C3 has also been described as an androgen receptor coactivator [36]. Our results indicate that the higher prostatic *AKR1C3* transcript levels in patients with T2D could be responsible for an increased androgen receptor level or activation, which was previously detected in the prostate glands of patients with T2D [37]. Although AKR1C1 and C2 are implicated in DHT catabolism, they are also involved in the regulation of oxidative stress and inflammation [30].

**Figure 5:**
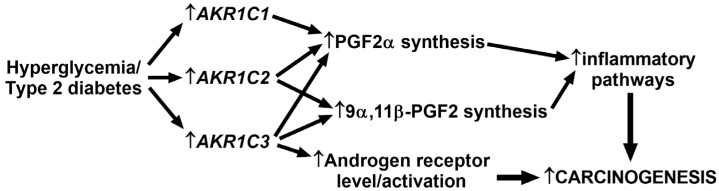
Proposed carcinogenic activity of the increased *AKR1C* transcript levels in prostate samples of patients with type 2 diabetes. AKR1C1, C2, and C3 enzymes are responsible for prostaglandin 2α (PGF2α) and 9α,11β-prostaglandin 2 (9α,11β-PGF2) synthesis. These prostaglandins can activate the NFκB pathways and inflammatory processes that drive carcinogenesis. Furthermore, AKR1C3 is involved in the synthesis of androgen receptor ligands (testosterone and dihydrotestosterone) and may also induce the transcript of androgen receptors. The elevated androgen receptor signaling has been described to induce carcinogenic processes in PCa.

In both PCa and T2D conditions, oxidative stress and inflammatory processes, including hypoxia (HIF1α) and NFκB pathways, play a crucial role [38]. Burczynski and colleagues observed that oxidative stress induced the expression of *AKR1C1* [39]. It is, therefore, conceivable that oxidative stress is responsible for the induction of *AKR1C1* transcript, in parallel with the activated HIF1α and NFκB pathways, in the prostate tissue of patients with T2D. In cancer cells, hypoxia (HIF1α) and inflammatory (NFκB) pathways are tightly interconnected via prostaglandin synthesis. Hypoxia has been shown to increase the level of prostaglandin 2 alpha (PGF2α), which is one of the main active prostaglandins [40]. Moreover, prostaglandins are the central regulators of inflammation-triggered carcinogenesis [41]. All three AKR1C members are involved in prostaglandin metabolism [41]. AKR1C enzymes can, for example, catalyze the production of PGF2α and 9α,11β-PGF2, both of which are potent activators of the NFκB pathway and inflammation [41,42]. Furthermore, in PC3 cells, overexpression of *AKR1C2* induced PGF2α level and *AKR1C2* expression is positively correlated with an increase in the Gleason score, and thus in disease progression in human prostatic cancer [43]. These results indicate that in prostate samples of patients with T2D, the higher *AKR1C* transcript levels may elevate the synthesis of bioactive prostaglandins, which, in turn, could induce the NFκB-inflammatory processes that lead to accelerated tumor progression (Figure 5).

The application of novel AKR1C3 inhibitors is characterized by promising anticancer effects since AKR1C3 inhibitors decreased prostate-specific antigen (PSA) expression and cell proliferation in LNCaP cells [36,44]. Moreover, drugs applied in the treatment of diabetes could exert their potential anticancer effects via the inhibition of AKR1C enzymes. Sulfonylureas, which increase insulin secretion in the beta cells, are still widely used for the treatment of T2D [6]. Sulfonylurea treatment of T2D patients showed conflicting results with regard to cancer risk [45]. Nevertheless, Yang and colleagues observed a lower cancer risk among Hong Kong Chinese patients with T2D who had been treated with glibenclamide and gliclazide sulfonylureas [46]. In-vitro experiments showed that glibenclamide inhibited the enzyme activities of AKR1C1, C2, and C3 [27]. These results suggest that inhibition of AKR1C activities may lower the cancer risk in T2D patients treated with glibenclamide. Further studies are required to clarify the role of AKR1C enzymes in the development of PCa. Nevertheless, our data indicate that targeting AKR1C enzymes in patients with T2D may be a potential new therapeutical approach.

## 4. Materials and Methods

Human samples

Newly-diagnosed PCa patients were recruited prior to radical prostatectomy. Tissue sampling was performed by an experienced uropathologist. PCa, as well as benign tissue, was immediately snap-frozen in liquid nitrogen and stored at −80 °C. For histological confirmation, hematoxylin and eosin staining was performed on paraffinized samples. Histopathological features were assessed, and postoperative Gleason scores were determined [47]. Patient cohorts were age- and BMI-matched, and *n* = 17‒17 benign and *n* = 11‒11 tumor tissue samples from patients with and without type 2 diabetes were included in this study (Table S1). Informed written consent was obtained from all participants, and the Ethics Committee of the University of Tübingen approved the protocol in accordance with the Declaration of Helsinki. Total RNA was isolated using an Allprep RNA/DNA/protein kit (Qiagen, Hilden, Germany) in accordance with the manufacturer´s instructions.

Cell culture

The human prostate adenocarcinoma cell line PC3 (CLS-Cell line services GmbH, Eppelheim, Germany) was originally isolated from bone metastasis of a patient with prostate cancer. Cells were maintained in low glucose DMEM (5.5 mM) supplemented with 5% fetal bovine serum (FBS). One day before treatment with increasing glucose concentrations, the medium was changed to DMEM low-glucose with 0.2% FBS. Following the addition of 5.5, 11.25, and 17.5 mM glucose, the cells were incubated for a further 72 h. Five independent experiments were performed. Following cell harvest, total RNA was isolated using an Allprep DNA/RNA/miRNA kit (Qiagen) in accordance with the manufacturer’s instructions.

Real-time PCR

From total RNA, cDNA was synthesized using the Transcriptor First Strand cDNA synthesis kit (Basel, Roche, Switzerland) [48]. Real-time PCRs were performed with LightCycler 480 Probes Master (Roche) and universal probe library or custom-made probes using LightCycler 480 (Roche), as published previously [49]. Delta–delta crossing-point (Cp) values were calculated, and the data were normalized to the housekeeping gene ubiquitin c (UBC) [50]. For real-time PCR analysis, the following primers and probes were applied: Aldo-keto reductase family 1 (AKR1) member C1 (C1) *AKR1C1* 5′-GGCAATTGAAGCTGGCT and 3′-AACTCTGGTCGATGGGAAT (probe: CAGGTTGGACTGGCCATCCGAAGC), *AKR1C2* 5′-GTAAAGCTCTAGAGGCCGT and 3′-AACTCTGGTCGATGGGAAT (probe: CAGGTTGGACTGGCCATCCGAAGC), *AKR1C3* 5′-GTAAAGCTTTGGAGGTCACAA and 3′-TCTGGTCGATGAAAAGTGG (probe: CAGGTTGGACTGGCCATCCGAAGC), *AKR1B1* 5′-GGGGTTGGGTACCTGGAA and 3′-GGTACCCGACGTCAATGG (probe nr 37), angiopoietin like 4 (*ANGPTL4)* 5′-GTTGACCCGGCTCACAAT and 3′-GGAACAGCTCCTGGCAATC (probe nr 44), glucose transporter type 1 (*GLUT1)* 5′-GGTTGTGCCATACTCATGACC and 3′-CAGATAGGACATCCAGGGTAGC (probe nr 67), hypoxia inducible factor 1 subunit alpha (*HIF1α*) 5′-GATAGCAAGACTTTCCTCAGTCG and 3′-TGGCTCATATCCCATCAATTC (probe nr 64), baculoviral IAP repeat containing 5 (*BIRC5)* 5′-CCGCATCTCTACATTCAAGAACT and 3′-GCCAAGTCTGGCTCGTTC (probe nr 43), glutathione peroxidase 2 (*GPX2)* 5′-CTGGTGGTCCTTGGCTTC and 3′-TGTTCAGGATCTCCTCATTCTG (probe nr 2), nitric oxide synthase 2 (*NOS2)* 5′-GCTCAAATCTCGGCAGAATC and 3′-GCCATCCTCACAGGAGAGTT (probe nr 42), RELA proto-oncogene, NFκB subunit (*RELA)* 5′-ACCGCTGCATCCACAGTT and 3′-GATGCGCTGACTGATAGCC (probe nr 47), suppressor of cytokine signaling 2 (*SOCS2)* 5′-GATCGCTATCCTTCCCTGAAC and 3′-AAGGGATGGGGCTCTTTCT (probe nr 65), marker of proliferation Ki-67 (*MKI67)* 5′-CCAAAAGAAAGTCTCTGGTAATGC and 3′-CCTGATGGTTGAGGCTGTTC (probe nr 39), proliferating cell nuclear antigen (*PCNA)* 5′-TGGAGAACTTGGAAATGGAAA and 3′-GAACTGGTTCATTCATCTCTATGG (probe nr 69), ubiquitin C (*UBC)* 5′-GGAAGGCATTCCTCCTGAT and 3′-CCCACCTCTGAGACGGAGTA (probe nr 11). For six genes, real-time PCR data from the PCa noT2D group have been previously published [48].

Statistical analysis

For the gene expression analysis of human data, Mann–Whitney tests were applied using GraphPad Prism 8.4 (USA). For the gene expression analysis of PC3 data, paired Friedman’s tests with Dunn´s multiple comparisons were calculated using GraphPad Prism. Data were logarithmic transformed prior to regression analyses. Multiple linear regression models were built using JMP 14.2 (SAS, Cary, NC, USA), and standard beta and *p*-values were calculated. For selected genes, leverage plots are shown. For human data, linear regression models were adjusted to age and BMI. Statistical significance was considered as *p* < 0.05.

## 5. Conclusions

Since the prostate tissue of patients with T2D *AKR1C1, C2,* and *C3* transcripts showed positive associations with proliferative, HIF1α, and NFκB genes, our results suggest that in the prostate gland, AKR1C enzymes could be involved in the induction of inflammatory processes induced by diabetes.

## Figures and Tables

**Table 1:** The gene expression levels of *AKR1C1*, *AKR1C2,* and *AKR1C3* were related to the genes indicated using multiple linear regression models, which were adjusted for age and BMI in benign prostate and PCa samples of PCa patients, both with and without type 2 diabetes (*n* = 56). Standard β values represent standardized regression coefficients. Significant gene associations are indicated in bold lettering, nonsignificant gene associations in italics. Statistical significance was considered as *p* < 0.05.

Linear Regression		*AKR1C1*		*AKR1C2*		*AKR1C3*	
Pathway	Gene	Standard β	*p*-Value	Standard β	*p*-Value	Standard β	*p*-Value
AKR1C	***AKR1C1***			**0.5122**	**<0.0001**	**0.6021**	**<0.0001**
	***AKR1C2***	**0.5524**	**<0.0001**			**0.4702**	**0.0003**
	***AKR1C3***	**0.6554**	**<0.0001**	**0.4746**	**0.0003**		
HIF1α	***ANGPTL4***	*0.2586*	*0.0683*	*0.2176*	*0.1125*	*0.0550*	*0.6903*
	***GLUT1***	*0.1761*	*0.2108*	*0.1155*	*0.3958*	*0.0790*	*0.5601*
	***HIF1α***	**0.4168**	**0.0015**	**0.3059**	**0.0175**	**0.4182**	**0.0008**
NFκB	***BIRC5***	*0.0548*	*0.6973*	*−0.0054*	*0.9683*	*0.2577*	*0.0522*
	***GPX2***	**0.3629**	**0.0086**	**0.2861**	**0.0334**	*0.0225*	*0.8693*
	***NOS2***	**0.2843**	**0.0410**	**0.4239**	**0.0011**	**0.3070**	**0.0207**
	***RELA***	**0.3817**	**0.0042**	*0.2190*	*0.0963*	**0.3734**	**0.0034**
	***SOCS2***	*−0.0692*	*0.6319*	*−0.0281*	*0.8399*	*−0.1760*	*0.2008*
proliferation	***MKI67***	*0.1604*	*0.2341*	*0.0340*	*0.7944*	*0.2485*	*0.0517*
	***PCNA***	**0.3425**	**0.0085**	*0.1419*	*0.2702*	*0.2245*	*0.0771*

**Table 2:** The gene expression levels of *AKR1C1, AKR1C2,* and *AKR1C3* were related to the genes indicated using multiple linear regression models in 5.5, 11.25, and 17.5 mM glucose-treated PC3 cells (*n* = 15). Standard β values represent standardized regression coefficients. Significant gene associations are indicated in bold lettering, nonsignificant gene associations in italics. Statistical significance was considered as *p* < 0.05. n.d.; mRNA level of *NOS2* was not consistently detected in PC3 cells.

Linear Regression		*AKR1C1*		*AKR1C2*		*AKR1C3*	
Pathway	Gene	Standard β	*p*-Value	Standard β	*p*-Value	Standard β	*p*-Value
AKR1C	***AKR1C1***			**0.9654**	**<0.0001**	**0.9582**	**<0.0001**
	***AKR1C2***	**0.9654**	**<0.0001**			**0.9773**	**<0.0001**
	***AKR1C3***	**0.9582**	**<0.0001**	**0.9773**	**<0.0001**		
HIF1α	***ANGPTL4***	**0.8545**	**<0.0001**	**0.8664**	**<0.0001**	**0.9181**	**<0.0001**
	***GLUT1***	**0.5896**	**0.0207**	**0.5393**	**0.0380**	**0.6683**	**0.0065**
	***HIF1α***	*0.1941*	*0.4883*	*0.2478*	*0.3732*	*0.3230*	*0.2403*
NFκB	***BIRC5***	**0.9595**	**<0.0001**	**0.9233**	**<0.0001**	**0.8979**	**<0.0001**
	***GPX2***	**0.9504**	**<0.0001**	**0.9053**	**<0.0001**	**0.8921**	**<0.0001**
	***NOS2***	n.d.		n.d.		n.d.	
	***RELA***	*−0.4090*	*0.1300*	*−0.5069*	*0.0538*	*−0.4840*	*0.0676*
	***SOCS2***	**0.6367**	**0.0107**	**0.5781**	**0.0240**	**0.5334**	**0.0406**
proliferation	***MKI67***	**0.9236**	**<0.0001**	**0.8583**	**<0.0001**	**0.8205**	**0.0002**
	***PCNA***	**0.8741**	**<0.0001**	**0.8843**	**<0.0001**	**0.8707**	**<0.0001**

## Supplementary Materials

The following are available online at https://www.mdpi.com/2075-4426/10/3/124/s1. Figure S1: Gene expression of *AKR1B1* in human prostate and PC3 samples; Table S1: Patient characteristics; Table S2: Gene expressions of proliferation processes and HIF1α and NFκB pathways in human prostate tissue.

## Abbreviations

ADT	Androgen deprivation therapy
AKR1	Aldo-keto reductase superfamily 1
BEN	Benign
BMI	Body mass index
CRPCa	Castrate-resistant prostate cancer
DHT	Dihydrotestosterone
HIF1α	Hypoxia-inducible factor 1 subunit alpha
NFκB	Nuclear factor of kappa light chain gene enhancer in B-cells
PCa	Prostate cancer
PGF2α	Prostaglandin 2 alpha
T	Testosterone
T2D	Type 2 diabetes
