# Supplementary material for: Transcript Levels of Aldo-Keto Reductase Family 1 Subfamily C (AKR1C) Are Increased in Prostate Tissue of Patients with Type 2 Diabetes
Source: J Pers Med. 2020 Sep 12;10(3):124. doi: 10.3390/jpm10030124 (PMC7564141; doi:10.3390/jpm10030124)
Supplement: Supplementary file 1 [file jpm-10-00124-s001.pdf]

Transcript levels of aldo-keto reductase family 1, subfamily C (AKR1C) are increased in prostate tissue of patients with type 2 diabetes

Andras Franko, Lucia Berti, Jörg Hennenlotter, Steffen Rausch, Marcus O. Scharpf, Martin Hrabě de Angelis, Arnulf Stenzl, Andreas L. Birkenfeld, Andreas Peter, Stefan Z. Lutz, Hans-Ulrich Häring, Martin Heni

Supplementary Materials

Table S1. Patient characteristics. Statistical significance was calculated among BEN, noT2D vs BEN, T2D as well as PCa, noT2D vs PCa, T2D samples using Mann-Whitney tests. Statistical significance was considered as $p<0.05$. BMI: body mass index; n: number of patients; BEN: benign prostate tissue; PCa: prostate cancer tissue.

**Parameter**	**BEN, noT2D** mean ± SD	**BEN, T2D** mean ± SD	**BEN** p-value	**PCa, noT2D** mean ± SD	**PCa, T2D** mean ± SD	**PCa** p-value
**Age [year]**	67 ± 5	63 ± 6	0.0765	65 ± 8	64 ± 3	0.7352
**BMI [kg/m^2]**	27.6 ± 3.3	29.5 ± 4.9	0.3940	26.5 ± 3.2	28.9 ± 3.8	0.1879
**Gleason 7a**	-	-	-	7	7	-
**Gleason 7b**	-	-	-	4	4	-
**n**	17	17	-	11	11	-

Table S2. Relative gene expression levels were measured in benign (BEN) prostate and prostate cancer (PCa) tissues of patients with (T2D) or without (noT2D) type 2 diabetes. Statistical significance was calculated among BEN, noT2D vs BEN, T2D as well as PCa, noT2D vs PCa, T2D samples using Mann-Whitney tests. Significant differences are shown in bold, non-significant differences are indicated in italic. Statistical significance was considered as $p<0.05$.

**Pathway**	**Gene**	**BEN, noT2D** mean ± SD	**BEN, T2D** mean ± SD	**BEN** p-value	**PCa, noT2D** mean ± SD	**PCa, T2D** mean ± SD	**PCa** p-value
HIF1α	***ANGPTL4***	*0.0152 ± 0.0164*	*0.0146 ± 0.0099*	*0.4332*	*0.0078 ± 0.0065*	*0.0100 ± 0.0101*	*0.8470*
	GLUT1	*0.0925 ± 0.0519*	*0.0932 ± 0.0557*	*1.0000*	*0.0842 ± 0.0327*	*0.0853 ± 0.0308*	*0.9873*
	HIF1α	*0.6066 ± 0.2650*	*0.7629 ± 0.3382*	*0.0730*	*0.8476 ± 0.2779*	*0.8516 ± 0.5967*	*0.1713*
NFκB	***BIRC5***	*0.0016 ± 0.0012*	*0.0013 ± 0.0010*	*0.6339*	*0.0036 ± 0.0020*	*0.0036 ± 0.0030*	*0.6994*
	GPX2	*0.0220 ± 0.0298*	*0.0139 ± 0.0101*	*0.7273*	*0.0091 ± 0.0110*	*0.0100 ± 0.0094*	*0.5190*
	NOS2	*0.0014 ± 0.0015*	*0.0032 ± 0.0047*	*0.1270*	*0.0212 ± 0.0563*	*0.0014 ± 0.0014*	*0.5943*
	RELA	**0.2805 ± 0.1127**	**0.3498 ± 0.1109**	**0.0232**	*0.3421 ± 0.1221*	*0.3573 ± 0.0509*	*0.9487*
	SOCS2	*0.0100 ± 0.0078*	*0.0065 ± 0.0056*	*0.2415*	*0.0069 ± 0.0083*	*0.0051 ± 0.0049*	*0.8977*
proli-	***MKI67***	*0.0146 ± 0.0122*	*0.0099 ± 0.0066*	*0.1932*	*0.0171 ± 0.0079*	*0.0281 ± 0.0228*	*0.3653*
feration	***PCNA***	*0.1108 ± 0.0574*	*0.0978 ± 0.0540*	*0.1932*	**0.0939 ± 0.0255**	**0.1155 ± 0.0259**	**0.0192**

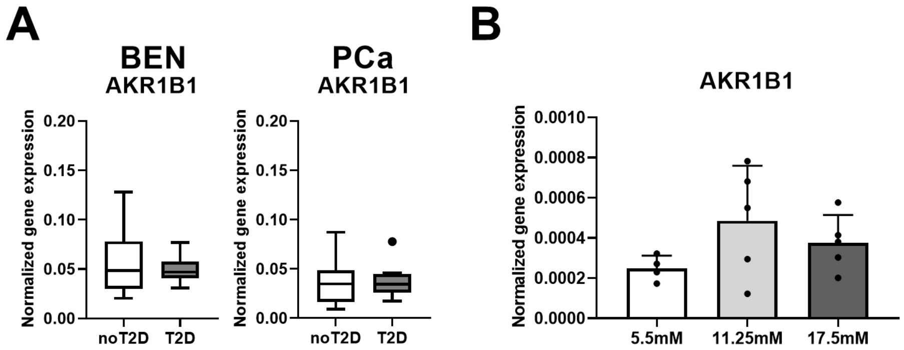

Figure S1.

A Gene expression of *AKR1B1* was measured in benign (BEN) prostate (n=17-17) and prostate cancer (PCa) (n=11-11) tissues of patients with (T2D) or without (noT2D) type 2 diabetes. Data are shown as Tukey box plots. Statistical significance was calculated among BEN, noT2D vs BEN, T2D as well as PCa, noT2D vs PCa, T2D samples using Mann-Whitney tests. **B** Gene expression of *AKR1B1* was measured in 5.5-, 11.25- and 17.5 mM glucose treated PC3 cells (n=5 independent experiments). Statistical significance was calculated using paired Friedman test with Dunn´s multiple comparison. Statistical significance was considered as $p<0.05$.
